# Supplementary material for: Molecular Mapping and Candidate Gene Analysis for GA3 Responsive Short Internode in Watermelon (Citrullus lanatus)
Source: Int J Mol Sci. 2019 Dec 31;21(1):290. doi: 10.3390/ijms21010290 (PMC6982186; doi:10.3390/ijms21010290)
Supplement: Supplementary file 1 [file ijms-21-00290-s001.zip › ijms-677614-supplementary/Supplementary Materials/Supplementary Figure 1.docx]

**Supplementary Figure S1:** Phenotypic characteristics of short internode length before and after GA_3_ application. (**A**) Long internode plants before and after GA_3_ application, after 5 days from the date of GA_3_ application. (**B**) Short internode plants before and after GA_3_ application, after 5 days from the date of GA_3_ application; (**C**) Long internode plants and (**D**) short internode plants at flowering stage without GA_3_.


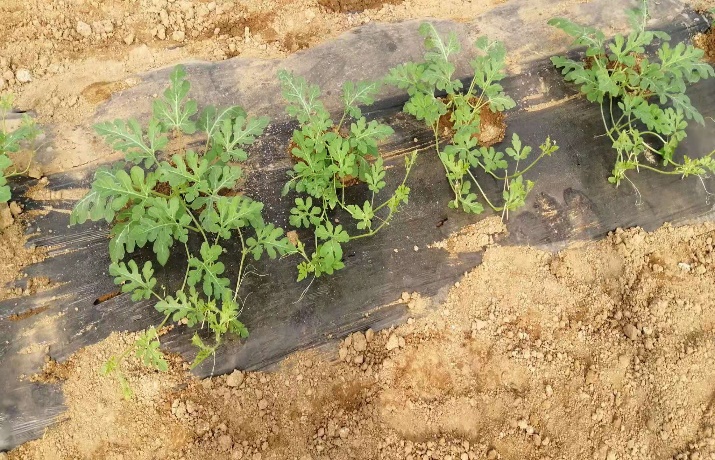

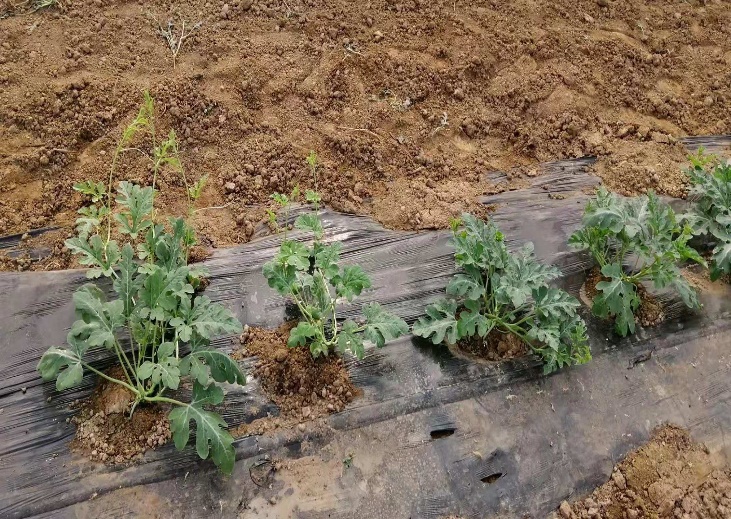


**A**

**LI**

**SI**

**SI+GA_3_**

**LI+GA_3_**

**B**

**C**


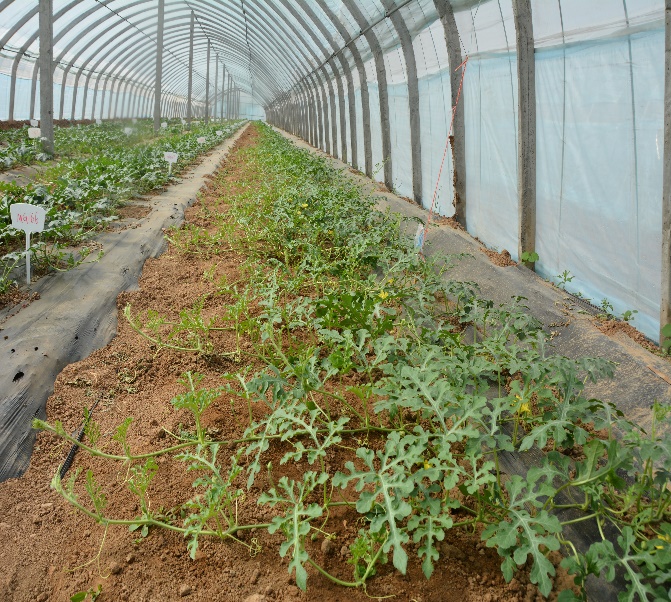

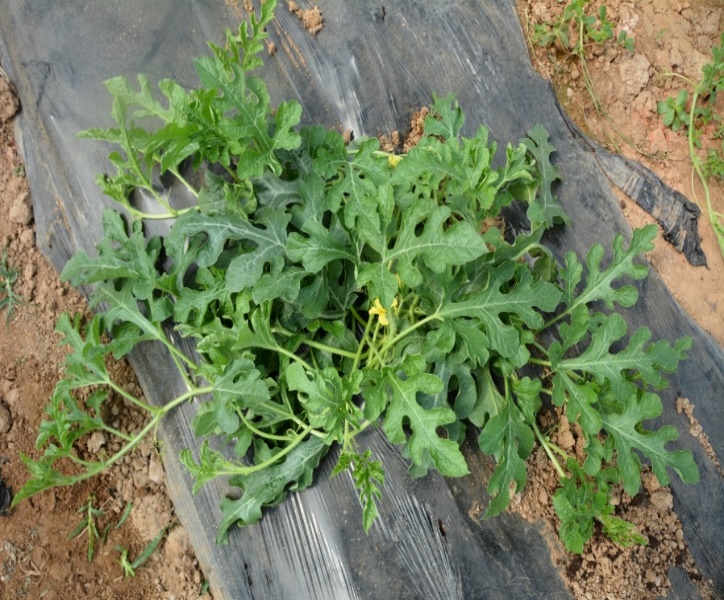


**SI**

**D**

**LI**
