# Supplementary material for: Molecular Mapping and Candidate Gene Analysis for GA3 Responsive Short Internode in Watermelon (Citrullus lanatus)
Source: Int J Mol Sci. 2019 Dec 31;21(1):290. doi: 10.3390/ijms21010290 (PMC6982186; doi:10.3390/ijms21010290)
Supplement: Supplementary file 1 [file ijms-21-00290-s001.zip › ijms-677614-supplementary/Supplementary Materials/Supplementary Table 1.docx]

**Supplementary Table S1**: The segregation ratio of short internode among populations (Zhengzhouzigua (P_1_) x Duan125 (P_2_))

| Generation | Population | Observed number of plants | | Segregation ratio | X^2^ | P-Value |
| --- | --- | --- | --- | --- | --- | --- |
|  |  | LI | SI |  |  |  |
| P_1_ | 50 | 50 | - | - | - |  |
| P_2_ | 50 | - | 50 | - | - |  |
| F_1_ | 22 | 22 | - | 1:0 | - |  |
| 2016 winter F_2_ Xinxiang | 367 | 278 | 89 | 3:1 | 0.110 | 0.740 |
| 2017 spring F_2_ Xinxiang | 430 | 325 | 105 | 3:1 | 0.078 | 0.781 |
| 2017 spring F_2_ Hainan | 578 | 431 | 147 | 3:1 | 0.058 | 0.81 |
| 2018 spring F_2_ Xinxiang | 486 | 367 | 119 | 3:1 | 0.069 | 0.79 |
| 2018 spring BC_1_P_1_ | 70 | 70 | - | 1:0 | - | - |
| 2018 spring BC_1_P_2_ | 80 | 38 | 42 | 1:1 | 0.20 | 0.198 |

P_1_= Zhengzhouzigua (Long internode), P_2_= Duan125 (Short internode), LI= Long internode, SI= Short internode. F_1_= individuals obtained by crossing Zhengzhouzigua with Duan125. BC_1_P_1_= F_1_ (Zhengzhouzigu x Duan125) x Zhengzhouzigua; BC_1_P_2_= F_1_ (Zhengzhouzigu x Duan125) x Duan125; F_2_ population derived from the self-pollination of F_1_ (Zhengzhouzigu x Duan125).
