# Supplementary material for: Molecular Mapping and Candidate Gene Analysis for GA3 Responsive Short Internode in Watermelon (Citrullus lanatus)
Source: Int J Mol Sci. 2019 Dec 31;21(1):290. doi: 10.3390/ijms21010290 (PMC6982186; doi:10.3390/ijms21010290)
Supplement: Supplementary file 1 [file ijms-21-00290-s001.zip › ijms-677614-supplementary/Supplementary Materials/Supplementary Table 3.docx]

Supplementary Table S3: Phenotypes and genotypes of recombinant individuals showing the recombinant breaking points

| **No.** | **CAPS56** | **CAPS85** | **CAPS90** | **CAPS91** | **CAPS106** | **CAPS107** | **CAPS108** | **CAPS123** | **CAPS142** | **CAPS148** | **Phenotype** |
| --- | --- | --- | --- | --- | --- | --- | --- | --- | --- | --- | --- |
| P1 | A | A | A | A | A | A | A | A | A | A | LI |
| P2 | a | a | a | a | a | a | a | a | a | a | SI |
| F1 | H | H | H | H | H | H | H | H | H | H | LI |
| 1 | a | a | a | a | H | H | H | H | H | H | SI |
| 7 | H | H | H | H | a | a | a | a | a | a | LI |
| 12 | A | H | H | H | a | a | a | a | a | a | LI |
| 22 | H | H | H | H | a | a | a | a | a | a | LI |
| 23 | A | H | H | H | a | a | a | a | a | a | LI |
| 38 | H | H | H | H | a | a | a | a | a | a | LI |
| 47 | H | H | H | H | a | a | a | a | a | a | LI |
| 60 | H | H | H | a | a | a | a | a | a | a | SI |
| 69 | a | a | a | H | H | H | H | H | H | H | SI |
| 71 | H | H | H | H | a | a | a | a | a | a | LI |
| 91 | H | H | H | H | a | a | a | a | a | a | LI |
| 153 | H | H | H | a | a | a | a | a | a | a | SI |

LI=Lon internode. SI= Short internode. Alleles were abbreviated according to their origin: A=Zhengzhouzigua (long internode); a=Duan125 (Short internode); H=heterozygous.
